# Cationic Polymerization of Hexamethylcyclotrisiloxane in Excess Water

**DOI:** 10.3390/molecules26154402

**Published:** 2021-07-21

**Authors:** Quentin Barnes, Claire Longuet, François Ganachaud

**Affiliations:** 1 Ingénierie des Matériaux Polymères, CNRS UMR 5223, INSA-Lyon, Univ Lyon, F-69621 Villeurbanne, France; quentin.barnes@hotmail.fr; 2 IMT—Mines Ales, Polymers Hybrids and Composites (PCH), 6 Avenue De Clavières, F-30319 Alès, France; claire.longuet@mines-ales.fr

**Keywords:** Piers-Rubinsztajn catalyst, surfactant-free polymerization, polydimethylsiloxane

## Abstract

Ring-opening ionic polymerization of cyclosiloxanes in dispersed media has long been discovered, and is nowadays both fundamentally studied and practically used. In this short communication, we show some preliminary results on the cationic ring-opening polymerization of hexamethylcyclotrisiloxane (D_3_), a crystalline strained cycle, in water. Depending on the catalyst or/and surfactants used, polymers of various molar masses are prepared in a straightforward way. Emphasis is given here on experiments conducted with tris(pentafluorophenyl)borane (BCF), where high-molar polymers were generated at room temperature. In surfactant-free conditions, µm-sized droplets are stabilized by silanol end-groups of thus generated amphiphilic polymers, the latter of which precipitate in the course of reaction through chain extension. Introducing various surfactants in the recipe allows generating smaller emulsions in size with close polymerization ability, but better final colloidal stability, at the expense of low small cycles’ content. A tentative mechanism is finally proposed.

## 1. Introduction

Silicones are polymers of broad interest, both on industrial and academic sides. In industry, developments focus on the generation of more and more performing elastomers, widely used in numerous applications, e.g., for their thermal resistance or their innocuity [1]. In academia, a great deal of work has recently been conducted on (mostly rediscovered) chemistries to generate silicone chains with new functionalities. In the most recent research, we can cite, in particular, aza-Michael addition [2], thiol-ene click chemistry [3], and organocatalyzed polymerization and polycondensation (see, e.g., [4]).

A recurrent domain of development concerns the generation of silicone aqueous emulsions via the ring-opening polymerization of cyclosiloxanes (a recent book published by the Dow company summarizes recent comprehension of silicone dispersions [5]). Both cationic and anionic catalysts produce silicone dispersions, but the mechanism by which long polymer chains are formed differs, and is still of debate nowadays [5,6]. Basically, taking the case of octamethylcyclotetrasiloxane (D_4_), polymerizations in emulsion (fast mixing in the presence of excess surfactant), in microsuspension (pre-generation of nanosized monomer droplets, typically by ultrasonication), or microemulsion (thermodynamically stable nanodroplets) follow very different pathways. In all instances, ring opening proceeds to propagate chains. Polymerization is stopped by transfer to water, but silanol chains ends can be reactivated to propagate further. Polycondensation between silanol end-groups also take place, to increase the molar masses. Side reactions that are generally observed in bulk or solution are likely prominent in water: backbiting that gives rise to a variety of cyclosiloxanes with various reactivities, and intermolecular redistributions between chains that enlarge the molar mass distribution. All the difficulty in these systems is to first understand where the different reactions take place (directly in water, at the droplet interface or inside the droplets), and second, at which paces.

Hexamethylcyclotrisiloxane is a monomer of choice when targeting silicone chains with perfectly controlled masses and functionality, as obtained by living anionic polymerization (for a very recent review, see an article just published in this Special Issue [7]). On the other hand, cationic polymerization of D_3_ has been described mostly in the mid-eighties by two groups led by P. Sigwalt and J. Chojnowsky, and not further exploited (a summary of this more than 10 year’s competition can be found in [8]). Using triflic acid as a superacid, the team of Sigwalt showed that water would retard the polymerization of D_3_, but would not inhibit it, albeit at a high catalyst content. To our knowledge, ring-opening cationic polymerization of D_3_ in water has hardly been studied. Early on, Weyenberg et al. showed, in a seminal paper, that D_4_ or D_3_ was easily converted into polymers in the presence of dodecylbenzenesulfonic acid [9]. They wrote that ‘*polymerization of hexamethylcyclotrisiloxane proceeds at a much faster rate than the cyclotetrasiloxane and, in fact, it is not necessary to pre-emulsify this monomer. Contact of even large crystals of this monomer with DBSA and water at 25 °C gave a quantitative conversion to emulsion polymer within 24 hr’*. Hemery et al. have later polymerized D_3_, solubilized in toluene, in emulsion by an anionic polymerization process, where they observed fast generation of polymers with an unlikely broad distribution [10].

Tris(pentafluorophenyl)boron (acronym BCF) was discovered in the early 1960s, and was almost forgotten for 25 years before being rediscovered as a catalyst activator in metallocene-catalyzed olefin polymerization. Its strong Lewis acidity, comparable to those of BF_3_.OEt_2_, combined with its air stability and water tolerance, has made it a (co-)catalyst of choice for numerous reactions that are summarized in reviews (e.g., [11]). Since the discovery by Piers et al. that BCF catalyzes the reduction reaction of a silyl ether into alkane in the presence of an hydrogenosilane, this reaction was later patented and published to produce linear silicone chains from alkoxy- and hydrogeno-functionalized silicone molecules [12]. The so-called Piers–Rubinsztajn reaction was studied in detail, particularly in the team of Professor Chojnowsky in a series of papers explaining the mechanism of catalysis. A precision reaction could thus be performed, starting from model molecules, allowing the generation of complex branched structures with exceptional monodispersity (for a review on this, please see [13]). For the record, this Lewis acid was also proved to promote a hydrosilylation reaction, albeit in stoichiometric amount, or, more strangely, oligomerization of electron-withdrawing monomers (typically vinyl methylsulfone or acrylonitrile) onto SiH functions through a coordinated ate-type intermediate [14]. The team of Chojnowsky has shown that D_3_ can be open and polymerized by tetramethyldisiloxane (L_2_H) and other hydrogenosiloxanes in BCF toluene solution, but polymerization does not proceed in the absence of these molecules [15].

In this communication, we propose to show some preliminary results on the cationic polymerization of D_3_ in excess water. Thanks to our deep knowledge on such processes applied to cyclosiloxanes [6] and vinyl monomers [16], we have selected a variety of Bronsted and (water-tolerant) Lewis acids. We particularly show advanced results on the polymerization of D_3_ using BCF as a catalyst, and finally propose a brief discussion about a tentative polymerization mechanism.

## 2. Results and Discussion

### 2.1. First Screening

**Figure 1:**
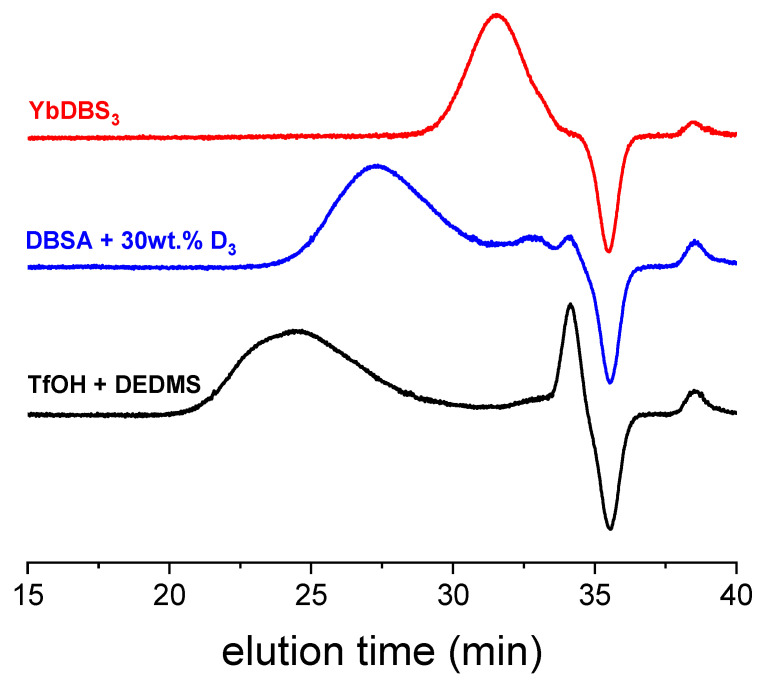
Selected SEC chromatograms of samples polymerized with various acids in the first set of polymeriations. Negative peak at 36 min is due to the flow marker. For the record, D_3_ and D_5_ were separately analyzed by SEC and came out at 34.5 and 34 min, respectively.

Table 1 summarizes the different results of D_3_ polymerization in excess water, using different catalysts. Basically, molar masses and polydispersity, as well as the final contents of polymer, are given here, with selected SEC traces plotted in Figure 1. All the experiments were conducted at room temperature, in 10 mL vials, using a magnetic agitation (see conditions in Table 1 footnote). We did not specifically look at the colloidal state of the dispersions here, nor did we follow the kinetics of the reaction.

The first experiments conducted with triflic acid in large quantities showed that solid D_3_ is rapidly consumed to give a totally transparent solution. We could not track the presence of polymer by precipitation of a sample aliquot in excess methanol, nor any other cycles that would have generated oil stains on the vial’s walls. After the addition of a slight quantity of diethoxydimethylsilane (DEDMS, 0.2 eq. of initial D_3_) and agitation during 12 h, we observed a precipitate at the bottom of the tube assigned to a polymer of high molar mass (M_n_ of 180,000 g/mol by SEC). DEDMS introduced in triflic acid aqueous solution in absence of D_3_ did not produce a polymer, in agreement with a previous study [17]. We then concluded that triflic acid opens the cycle to generate water-soluble oligomers that convert into polymer by acid-catalyzed condensation between silanol and ethoxysilane groups. Note that this reaction is very different from the polycondensation reaction of bis-silanol-terminated PDMS long, hydrophobic oligomers that occurs exclusively at droplet interfaces [18].

Changing a molecular superacid to an acidic surfactant, DBSA, after only 6 h of reaction, we could detect the presence of polymers in the test tubes. SEC curves give molar masses of around 70,000 g/mol, with a larger content of small cycles at a larger D_3_ content (about 10 wt.% at the end of the polymerization). Since at the time we were looking for cycle-free emulsions, we did not further explore this path; we are currently pursuing some experiments to check how fast and efficient this polymerization is.

We also tested some rare earth Lewis acids (ytterbium and indium chloride salts) combined with sodium dodecylbenzene sulfonate (NaDBS), to generate so-called Lewis acid surfactant complexes (LASCs), as reported before [16]. Even in large excesses, as tested here, these catalysts produced exclusively oligomers of molar masses around 4500 g/mol. Note that methanol also precipitated the LASC catalyst, so that it cannot be easily separated from the oligomers. According to the price of the catalysts used here, and the short oligomers produced, this alley was not pursued.

### 2.2. The Case of BCF

The origin of this second set of experiments comes from a study of the condensation reactions of alkoxy- and silanol-functionalized telechelic polymers in water [17]. When starting from the model molecules, tetramethyldisiloxane and dimethyldimethoxysilane, we observed, the rapid generation of cyclosiloxanes of various sizes, from D_3_ to D_7_, together with some polymer. The former cycle gradually disappeared with time, whereas the larger ones would accumulate in the reactor. This intriguing observation prompted us to further study the cationic ROP of D_3_ in water catalyzed by BCF, in the absence of any other siloxane- or silane-based molecules. Note that we preliminary checked that BCF does not promote D_3_ polymerization in toluene overnight (not shown).

A typical experiment consisted of introducing D_3_ powder straightaway in a test-tube containing an aqueous solution of the catalyst, at room temperature and under magnetic agitation (see formulation in Table 2, entry 5).

**Figure 2:**
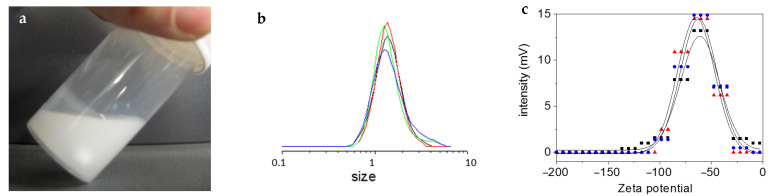
Colloidal state of D_3_ suspension polymerization: (**a**) metastable emulsion, where polymer stains can finally be seen on the top of the wall; (**b**) average particle size at different times of reaction to highlight the emulsion stability (every 15 min in the following order: red, black, green, blue); (**c**) zeta potential of emulsion after 6 h on three different samples (average value of −65 ± 15 mV).

Even if D_3_ first resides as solid chunks at the top of the water phase, it is then gently incorporated with time. A white emulsion (Figure 2a), of about 1.5 μm in size (Figure 2b), is generated, and remains almost so until the end of the reaction (we can track a slight enlargement in the size distribution with time, see Figure 2b). Zeta potential measurements give surface values of about –65 mV (Figure 2c). We suspect that the silanol groups of the oligomers/polymers protruding at the surface of the droplets stabilize them, as proposed earlier by Vincent et al. [19] and ourselves [17]. Only when the content of the silanol groups becomes too small that the polymer precipitates and deposits on the flask wall (Figure 2a).

**Figure 3:**
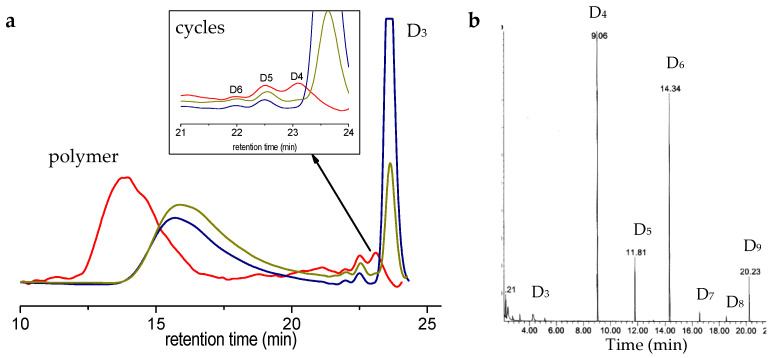
(**a**) SEC chromatograms of samples taken after ½ day (blue curve), 1 day (green olive curve) and 3 days (red curve). Zoom on the zone of small cycles is given on the inset. (**b**) GC-MS analyses of D_3_/BCF system after 12 h of reaction. Abundances are relative, since peaks appear larger as molar mass of the cycle increases.

Typical SEC traces are given in Figure 3a, along with interpretations of it; the average molar masses are reported in Table 2 at two different reaction times. It can be seen here that polymerization of D_3_ occurs quite smoothly, generating polymers of very high molar mass (typically 150,000 g/mol). This is typical of cationic polymerization in emulsion of cyclosiloxanes [6]. We were not capable of characterizing the chain-end of the polymers of such high molar masses; however, the fact that molar mass increases with time let us think that the polymers chains do not close ends here. Similar results were observed for the system starting from linear precursors [17].

We can also track, on the SEC trace, a rapid generation of small cycles (typically D_5_ and above) which contents grow slowly with time. Intermediate macrocycles are visible on the SEC trace after 3 days of reaction, showing that backbiting and intermolecular redistribution are retarded, but occur in this polymerization system (Figure 3a). To gain better insight into the course of polymerization, we have injected the intermediate sample in a GC/MS apparatus (Figure 3b). D_6_ and D_9_ appear together in larger proportions than other cycles, except for D_4_. We observed a similar accumulation of D_3x_^F^ cycles building (x = 2,3,4) in the anionic polymerization of D_3_^F^ in miniemulsion, before a backbiting reaction occurs extensively and generates intermediate cycles (D_4_^F^, D_5_^F^…), but no macrocycles [20].

### 2.3. Introducing Surfactants in the Recipe

**Figure 4:**
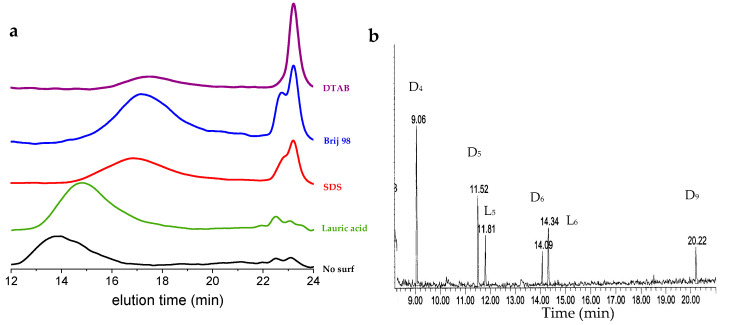
(**a**) SEC traces of different trials done in presence of surfactant, compared to pristine one. For polymerization conditions, see Table 2; (**b**) GC-MS analyses of D_3_/BCF/Brij system after 12 h of reaction.

As we noted before, the simplest system described above is heterogeneous, i.e., micron-sized droplets are slowly converted into a polymer film, precipitating on the walls of the test tube as a function of time. We tried to use different surfactants to ensure a stabilization of the dispersion throughout the process, while still carrying out the polymerization. Table 2 summarizes the different trials we made, and Figure 4 shows the corresponding SEC traces.

Using lauric acid, a translucent dispersion, typical of a microemulsion state, was obtained (average droplet size of 30 nm measured by DLS, not shown). Polymerization appeared to be quite fast, but led to the formation of larger contents of D_4_ and D_5_ than without a surfactant (more than 10 wt.%). Lauric acid also allowed rather large molar masses to be produced, around 55,000 g/mol, while keeping the colloidal stability of the dispersion.

With SDS, a similar microemulsion was formed, and polymerization was likely faster than without a surfactant. A polymer of a molar mass of around 15,000 g/mol was produced, together with a large load of small cycles (almost 30 wt.%). The same polymolecularity as for the other rounds was observed, typically around two. DTAB did not produce stable dispersions, and reactions led to a rapid generation of a large load of D_4_ (above 60%), together with polymers of a low molar mass (13 kg/mol). The fact that only D_4_ is generated here was not expected, and remains unexplained.

Not shown here is a trial with PVA, where an emulsion was formed, but polymerization did not proceed because of complexation of BCF with the alcohol groups of the dispersant. Brij 98 also complexes the catalyst through the oxygen atoms from ethylene glycol, but does not inhibit polymerization. Molar masses increase with time, together with the content of small cycles from D_4_ to macro ones. Looking at the GC/MS of the sample taken after 12 h (Figure 4b), we can notice the presence of tentatively assigned linear disilanol oligomers, in addition to the same cycles observed before (D_6_ and D_9_). This confirms the formation of molecular intermediates before they cycle back.

### 2.4. Proposed Polymerization Scheme

D_3_ polymerizes via a cationically catalyzed process in excess water and at room temperature. The initial screening showed that both Bronsted acids and Lewis acids catalyze the reaction, albeit at different paces and for final end results. The fact that triflic acid opens the cycle into small water-soluble oligomers, but does not convert them into polymers, seems to indicate that a condensation reaction of silanols is not likely in these conditions. This is certainly due to the absence of an interface, where this reaction generally takes place [6,18]. This also seems to confirm that, in contrast to the previously proposed emulsion polymerization of cyclosiloxane [5], small hydrophilic oligomers do not chain-extend in water. It would be worth in the future to mix together D_3_, DEDMS, and a non-ionic surfactant from the first place, with a view of generating a stable latex while gaining high molar mass silicone polymer.

DBSA and BCF catalysis holds the following comparable features: fast polymerization, large molar mass polymers, and a fair load of small cycles, as expected from such cyclosiloxane cationic process. This most likely shows that BCF acts here principally as a Bronsted catalyst. Ring opening, one to two condensation steps, and back-cyclization, together with true ring-opening polymerization, take place here. When adding lauric acid to the BCF system, an acidic surfactant that is too weak to participate to the reaction, the results match perfectly with the DBSA-catalyzed ones, as follows: molar masses of typically 55 to 70 kg/mol, stable dispersions and cycle contents of 10 wt.%. It would be interesting to introduce both DBSA and BCF in the recipe, to see if synergy occurs; we plan to conduct such process soon.

**Figure 5:**
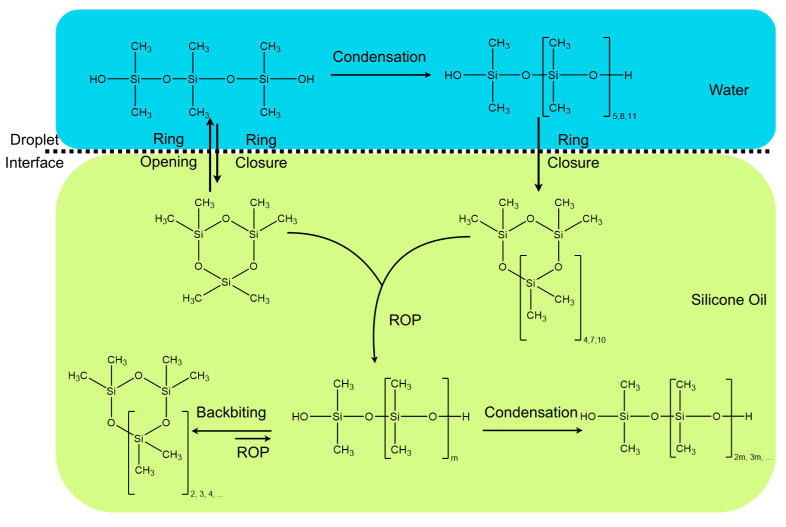
Proposed generic mechanistic scheme for the BCF-catalyzed D_3_ cationic polymerization in excess water. Here, the catalyst was omitted for sake of clarity.

Introducing other surfactants that could interact together with BCF led to more complex results. In all the cases, larger contents of cycles were observed, and polymerization was generally faster than in the absence of surfactants. Molar masses were rather low, around 15 kg/mol, which may be due to the larger content of water inside the monomer droplets, due to the presence of excess D_4_ (this cycle is more polar than silicone chains). The type of dispersions depend on the content and nature of the surfactant, but this was not studied in detail here. Figure 5 summarizes the reaction taking place in this process for the exemplary case of BCF.

## 3. Materials and Methods

Hexamethylcyclotrisiloxane (D_3_, 95%) was either kindly given by Bluestar Silicones (1st set of experiments) or purchased from ABCR (2nd set of experiments). Triflic acid (reagent grade, 98%), 4-DBSA (mixture of isomers, >95%), DTAB (>98%), SDS (98%) and lauric acid (≥98%) were all purchased from Sigma-Aldrich (Saint Louis, MO, USA). Brij 98 (hydroxyl titration: 50 to 65 mg KOH/g) came from ACROS organics (The Hague, Netherlands). Tris(pentafluorophenyl)borane (B(C_6_F_5_)_3_, purity 97%) was obtained from Lancaster (Watd Hill, MA, USA).

Here, two series of experiments were performed at different times and locations. In the first set of experiments, size exclusion chromatography, SEC, was carried out using a Malvern Viscotek (Malvern, UK) GPC Max apparatus equipped with three Shodex columns (KF-804, -805, and -806). Detection systems were a refractive index and differential viscometry detectors. Toluene (HPLC grade, provided by Sigma-Aldrich) was eluted at 1 mL/min with diisopropylethylamine as flow marker [21]. In the second set of experiments, a Spectra Physics (Andover, MA, USA) apparatus with two PL gel columns (5 µm particles size, 300 mm length, with the following two pores sizes: one with 50 Å and one with 100 Å) and a Styragel HR2 column (7.8 mm internal diameter × 300 mm length) were used. An SP8430 differential refractometer achieved the detection. The toluene was eluted at a flow rate of 0.8 mL/min using diethylether as a flow marker. In both systems, the temperature for the SEC column set and the detector chamber was 35 °C to ensure stable baselines, high chromatographic efficiency, and consistent results. The standards used to calibrate the SEC were polystyrene standards.

Gas chromatography coupled with a mass spectrometer (GC/MS) was done on a 6890 N apparatus from Agilent Technologies (Santa Clara, CA, USA), equipped with an electrospray mass detector Agilent 5973 N and an apolar capillary column HP5-MS 30 m × 0.25 mm (stationary phase made of a film of diphenyldimethylpolysiloxane 5%, 0.25 µm). Conditions used were as follows: initial temperature 45 °C during 2 min, temperature ramp of 2 °C/min up to 50 °C then 10 °C/min up to final temperature, 250 °C, set during 10 min. Peak integration were corrected with factors inherent of each silicone species, according to the procedure published elsewhere [22].

Particle sizes were determined by dynamic diffraction of a laser beam on a Nanotrac NPA 250 device (Microtrac Inc., Montgomeryville, PA, USA), typically in a size range between 8 nm and 6.54 µm. The light dispersed by the particles entails a Doppler effect, due to Brownian motion. The Microtrac^®^ Windows Software amplified, filtered, and mathematically treated this signal is to produce a size distribution.

## 4. Conclusions

To summarize, D_3_ is the cyclosiloxane of choice to generate silicone dispersions of very high molar mass polymers, a priori not reachable neither by conventional polycondensation in microsuspension nor emulsification. The fact that this monomer performs polymerization in almost all the catalytic systems screened here opens large avenues in the search for the ideal polymerization process that would hopefully not produce small cycles, such as D_4_ and D_5_, now targeted in the ‘Registration, Evaluation and Authorisation of Chemicals’ (REACh) European regulation.

## Figures and Tables

**Table 1:** First round of experiments of D_3_ cationic polymerization in excess water ^a^.

Exp.	D_3_Content (wt.%)	Catalyst	Cat.Content (wt.%)	Reaction Time (h)	M_n_ (kg/mol)	Đ	Conv. D_3_ (%)	Yield Polym. (%)
entry 1	30	TfOH	3	12	-	-	100	0
				12 (After DEDMS addition) ^b^	180	2.2	100	100
entry 2	15	DBSA	1	6	75	1.5	95	85
	30		1	6	63	1.6	90	80
entry 3	25	YbDBS_3_^c^	16	12	4.5	1.9	100	N.D. ^e^
entry 4	25	InDBS_3_^d^	15	12	4.5	1.9	95	N.D. ^e^

^a^ Typical procedure: all ingredients are mixed at once in 3 g water into 10 mL vial and a magnetic stirrer. After drying at 90 °C, samples were recovered in toluene and analyzed by SEC; acronyms: TfOH: triflic acid; DBSA: dodecylbenzenesulfonic acid; DEDMS: diethoxydimethylsilane; ^b^ addition of 5 wt.% of DEDMS; ^c^ prepared by mixing 3 mol eq of NaDBS with 1 eq of YbCl_3_.6H_2_O; ^d^ prepared by mixing 3 mol eq of NaDBS with 1 eq of InCl_3_; ^e^ LASCs coprecipitate with polymer, so it is not possible to calculate a yield in polymer.

**Table 2:** Second round of experiments of D_3_ cationic polymerization in excess water ^a^.

Exp.	Surf. ^b^	Cont. (wt.%)	State of Dispersion ^c^	Reaction Time (h)	M_n_ (kg/mol)	Đ	Yield Polym. (%)	Cycles but D_3_ (wt.% Content)
entry 5	-	-	H	12	26	2.0	56	D_5_ and above (41)
				72	144	2.2	89	D_4_, D_5_, D_6_ (7)
entry 6	Lauric acid	0.1	µE	12	51	2.4	88	D_4_, D_5_, D_6_ (11)
entry 7	SDS	0.1	µE	12	14.4	2.2	73	D_4_, D_5_ (27)
entry 8	Brij 98	0.5	E	6	3	1.4	47	D_6_ (3)
				12	25	2.3	65	D_4_, D_5_ and above (35)
entry 9	DTAB	0.1	H	12	13	1.5	39	D_4_ (61)

^a^ Typical procedure: all ingredients are mixed at once into a 10 mL vial equipped with a magnetic stirrer. Water = 5 g; D_3_ = 0.5 g (10 wt.%); BCF = 24 mg (0.5 wt.%). Samples are precipitated in methanol, dried, recovered in toluene and analyzed by SEC. ^b^ Acronyms: SDS: sodium dodecyl sulfate; DTAB: dodecyl trimethyl ammonium bromide; ^c^ H: heterogeneous; µE: microemulsion; E: emulsion.

## Data Availability

The data presented in this study are available on request from the corresponding author.

## Sample Availability

Samples are not available.
